# The Treatment of Fractures from a Common-Sense Point of View

**Published:** 1907-10-19

**Authors:** 


### Points in Surgery.

### THE TREATMENT OF FRACTURES FROM A COMMON-SENSE POINT OF VIEW.

### IX. FRACTURES IN THE NEIGHBOURHOOD OF THE ELBOW.

For a complete understanding of the fractures occurring in this situation a more or less intimate knowledge of the anatomy of the part is essential. The lower end of the humerus ossifies from four centres: that for the capitellum appears in the third year, for the inner condyle at the fifth, for the trochlea at the tenth, and for the external condyle at the fourteenth. The centres for the capitellum, trochlea, and external condyle fuse into one before joining with the shaft; this happens at the seventeenth year. The internal condyle joins the shaft separately a year or two later. The upper part of the capsule of the elbow-joint is intimately connected with the humerus. In front it is attached by a line which goes from the internal to the external condyle in a direction convex upwards, so that the coronoid fossa lies entirely inside the capsule of the joint. Behind its line of attachment is higher on the inner than on the outer side, extending from the inner condyle nearly at the top of the olecranon fossa obliquely downwards and outwards across the fossa towards the capitellum. A fracture of the internal condyle is far more likely to involve the joint than a corresponding fracture of the external condyle.

#### Transverse Supra-condyloid Fracture.

The humerus may be transversely fractured above the condyles. The diagnosis is usually obvious owing to the characteristic displacement. The lower fragment is drawn backwards behind the upper by the triceps muscle. Its lower end is also tilted backwards. This injury occurring in a muscular adult was mentioned in a previous article as one suitable for treatment by the operation of wiring; not because it is difficult to reduce the displacement, but because the retention of the fragments in apposition by a splint is a matter requiring great ingenuity. The best form of splint, in the writer's experience, is a posterior angular splint formed of two troughs of bent metal. These are connected by an external hinge at the elbow in such a manner that, when the arm is extended, the normal carrying angle of the limb is maintained. The splint can be fixed at any angle by means of a strong screw, which controls the hinge at the outer side of the elbow. The splint is well padded and applied to the arm with bandages. The arm is then flexed as far as possible and fixed in that position. The flexion serves to force the lower fragment into its proper position and to keep it there. It is well to take the arm out of the splint after three days and examine the position of the fragment under the *x*-rays. Massage and passive movement can be begun at the same date. The flexion can then be gradually reduced by altering the angle at the elbow. In three weeks the arm may be worn in a sling only.

#### Separation of the Lower Epiphysis of the Humerus.

Young patients, particularly between the ages of fifteen and seventeen, the period when the various centres for the lower end of the humerus have fused together but have not joined the shaft, if exposed to injury to the elbow, more often suffer from separation of the lower epiphysis. Without the *x*-rays this is more difficult to diagnose, because the lower fragment is so much smaller than in the former case that the displacement is less characteristic. In most cases all that can be felt is the lower end of the shaft protruding forwards in the front of the elbow. It is an almost invariable rule that when an epiphysis is separated by violence the shaft of the bone is displaced towards, and tends to press upon the main vessels of the limb. As in all cases of separated epiphysis, accurate coaptation is essential, because faulty union will interfere with the subsequent growth of the bone, and so lead to shortening of the limb. A serious complication in treatment is the fact that since the line of separation lies inside the capsule of the joint, there is nearly always much effusion and swelling. This is sometimes so great that no manipulation is possible for some days. If this is so, the patient should be put to bed with the arm in a poroplastic gutter splint—that is a splint made out of poroplastic to fit the limb roughly—and covered with an evaporating lotion to reduce the effusion. The following is an excellent lotion for the purpose: Ammonii chloridi ℥j., acidi acetici diluti ʒij., spiritus rectificati ℥ij., aquam distillatam ad Oj. Gentle massage also helps to reduce the effusion. When this object has been attained an anæsthetic should be given, the displacement carefully reduced, and the arm flexed if possible to its full extent, so that the hand can be bandaged down on to the shoulder of the affected side. If full flexion is impossible, the tin splint described above will be found useful, because by its means the amount of flexion can be increased daily. Early passive movement is important, so as to avoid limitation of movement at the joint.

#### Fractures Involving the Joint.

Various forms of fracture of the lower end of the humerus involving the elbow-joint itself are described, but are, fortunately, not common—fortunately because they are exceedingly difficult to treat successfully. Such are oblique fractures extending from the region of one or other condyle into the joint or a T-shaped fracture of the lower end of the bone. In the latter the humerus is transversely fractured at or about the level of the condyles, and the lower fragment is again broken into two or more pieces. After such an injury there is evidence of severe damage to the elbow, which is said to feel like "a bag of bones" to the examining finger. The condyles are broader than on the sound side. In these cases it is almost hopeless to attempt to obtain a good result with retentive apparatus and movement. If the attempt is made an anterior or posterior angular splint is the most satisfactory. Some stiffness of the joint nearly always results. If it seems likely, owing to the severity of the damage, that there will be little or no movement, the arm should be fixed at a right angle with the hand halfway between pronation and supination, this being the most useful position for all ordinary purposes. The results of operative treatment are hardly less disappointing. If operation be decided on, the arm should, of course, be thoroughly examined with the $x$-rays, so that the position of each fragment in relation to the others and to the shaft is known exactly. Access may be obtained by a posterior incision through which the triceps may be split longitudinally without separating the fibres from their insertion; or by lateral incisions. The former is the better; it sometimes happens that if lateral incisions are made and the tissues freely separated from the humerus round the condyles, the nutrition of the fragments is impaired. In artificially fixing portions of bone which are largely composed of articular surfaces the ordinary silver wire cannot be used. The best method to employ is to unite the fragments with an ivory peg, which is cut off flush with the surface and left *in situ*. These fractures are often compound on account of the extreme violence producing the injury, and when this is the case sepsis frequently supervenes, in spite of vigorous measures to prevent it. If the joint is infected it must be freely drained through adequate incisions; but a stiff joint nearly always results.

In the case of an immovable joint, the only treatment is excision of the elbow at a later date. It is an easy and successful operation. But the choice must be left with the patient after the facts have been explained to him. It is quite likely that if the arm is fixed in a useful position—that is at a right angle with the hand halfway between pronation and supination, he will prefer to remain as he is.

